# Navigation in Contour-Drawn Scenes Using Augmented Reality

**DOI:** 10.1177/20416695221074707

**Published:** 2022-01-31

**Authors:** Tadamasa Sawada, Alejandro Mendoza Arvizu, Maddex Farshchi, Alexandra Kiba

**Affiliations:** School of Psychology, 68192 HSE University, Moscow, Russian Federation

**Keywords:** navigation/wayfinding, augmented reality, contour-drawing, line-drawing, 3D perception, head-mounted display (HMD), scene perception

## Abstract

The visual system can recover 3D information from many different types of visual information, e.g., contour-drawings. How well can people navigate in a real dynamic environment with contour-drawings? This question was addressed by developing an AR-device that could show a contour-drawing of a real scene in an immersive manner and by conducting an observational field study in which the two authors navigated in real environments wearing this AR-device. The navigation with contour-drawings was difficult in natural scenes but easy in urban scenes. This suggests that the visual information from natural and urban environments is sufficiently different and our visual system can accommodate to this difference of the visual information in different environments.

Almost all studies in vision science are conducted in well-controlled laboratory environments that allow us to test specific factors while other, artifactual factors, are eliminated or minimized. But when this is done, it may become difficult to discuss whether, or how much, the factor tested will be critical in a real environment. The factor may affect a human's performance of some task in a laboratory experiment, but this factor may not be useful for doing the task in the real environment.

There are some field studies that used head-mounted prism goggles to test the performance of the visual system in a real environment (see Yoshimura, 1996 for a review). Prisms and mirrors in these goggles optically transform a retinal image in a real scene.

Note that image-filters of computer vision can be also used to transform retinal images by using Augmented-Reality (AR) technology (Anstis, 1992; Bao & Engel, 2019; Grush et al., 2015; Juan & Calatrava, 2011; Krösl et al., 2020; Velázquez et al., 2015). We developed an AR-headset that can show a contour-drawing generated by extracting luminance-edges in an image using a Sobel filter, or a grayscale-image of a real scene in an immersive manner (Farshchi et al., 2021). We showed that observers can perform some run-of-the-mill tasks almost equally well with both contour-drawings and grayscale-images (see Cole et al., 2009; Elder, 2018; Hertzmann, 2021; Pizlo et al., 2014; Sayim & Cavanagh, 2011). These tasks were performed by hands on a desktop while the observer was sitting still on a chair.

The authors (TS, AM) conducted this observational field study in which they wore the AR-headset and dynamically navigated in three real environments, namely, a forest, a park, and inside a building. The AR-headset showed a contour-drawing, a grayscale-image, or a color-image of a real scene to an observer in an immersive manner (Figure 1, see Farshchi et al., 2021 for details). The observers wore the headset intermittently to make it possible to change the image filters while they navigated.

**Figure 1.**
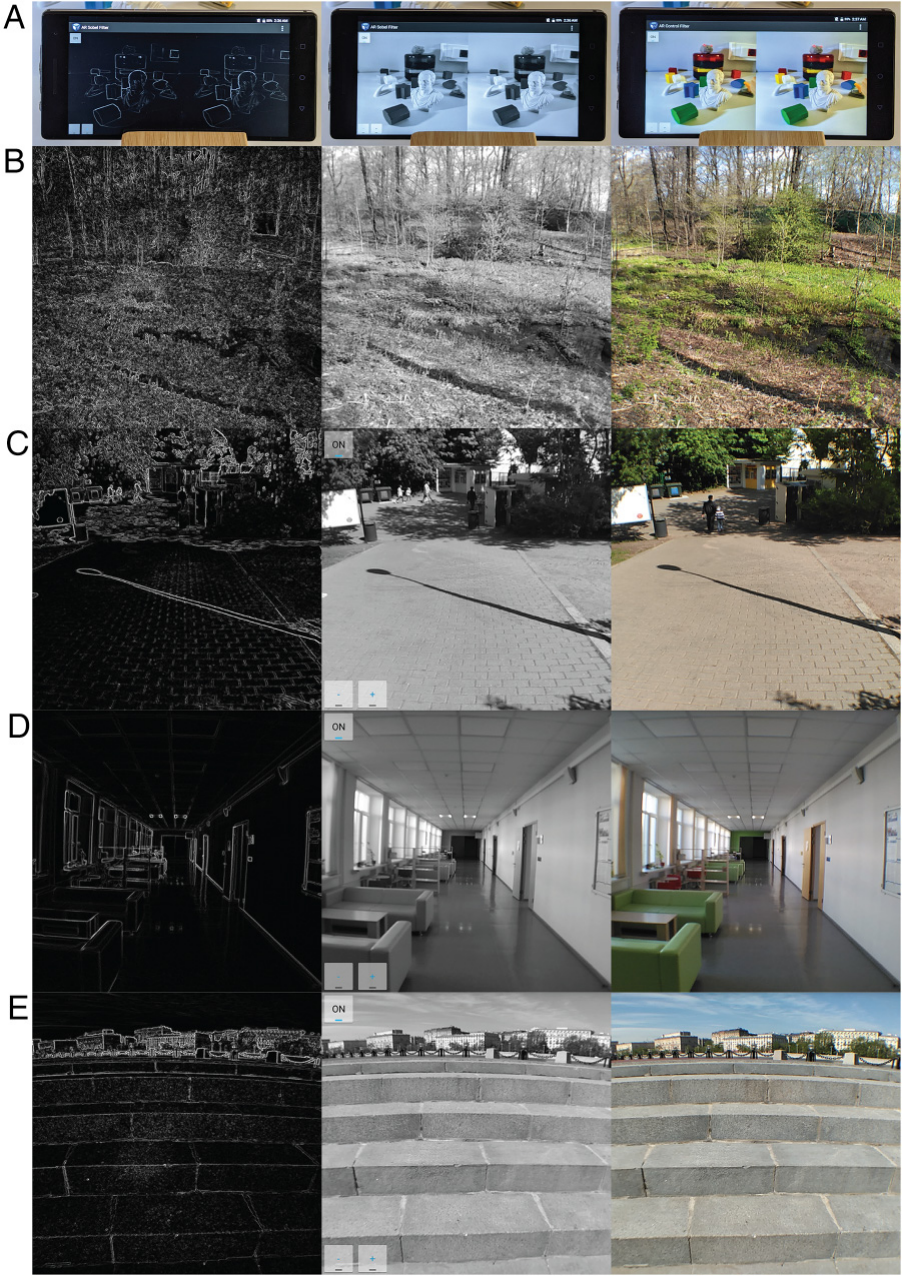
(A) A smartphone composing the AR-headset and (B-E) contour-drawings (left), grayscale-images (center), and color-images (right) of real scenes. Images are owned by the author TS. See https://osf.io/3tf9d/ for other images from this study.

Navigating in the real environment was always easier with the color- and grayscale-image filters than with the contour-drawing filter. Navigation with the contour-drawing filter was especially difficult in the forest. It was very difficult to see the bumps, dents, steps, and the slopes of the ground in the forest with the contour-drawing filter (Figure 1(B)). The information lost in a contour-drawing, for example, luminance-gradients and luminance-polarity of the shading and cast-shadows can be important in natural scenes.

Cast-shadows were also disturbing with the contour-drawing filter. The boundaries of these cast-shadows were represented as bright contours in the contour-drawings (Figures 1(B-C), Metzger, 1936/2006; Sayim & Cavanagh, 2011). The cast-shadows were often misperceived as markings on objects, or as objects, or crevasses on the ground.

Navigation was easy on the sidewalks and on the pavement in the park and in the hallways of a building even with the contour-drawing filter (Figures 1(C-D)). Subjectively, the curbs of the sidewalk, the regular pattern of the pavement, and the edges of the floor, walls, and ceiling of the hallways were helpful during the navigations, but note that stairs were very difficult to navigate with all the image filters, especially with the contour-drawing filter (Figure 1(E)). With the contour-drawing filter, the edges of the stairs were visible but it was hard to make out which faces of the stairs were horizontal or vertical.

Navigation was easy with the contour-drawings in the urban scenes. The urban scenes were composed of man-made objects and they were regular. These regularities of the scenes introduced configurations of contours that are model-based invariants of the regularities and that can be used to recover 3D information in the scenes by making use of the regularities as *a priori* constraints (Qian et al., 2018; Sawada et al., 2015; Walther & Shen, 2014).

Our study suggests that visual information available in natural and urban environments is quite different and our visual system can accommodate to this difference of the visual information in different environments.
